# Microstructure and Properties of Ag-SnO_2_ Electrical Contact Composites with Different SnO_2_ Volume Fractions

**DOI:** 10.3390/ma19163545

**Published:** 2026-08-21

**Authors:** Zhijie Lin, Bin Liu, Xudong Sun

**Affiliations:** 1 College of Materials Science and Engineering, Fujian University of Technology, Fuzhou 350118, China; 2 School of Materials Science and Engineering, Northeastern University, Shenyang 110819, China; 3 Foshan Graduate School of Innovation, Northeastern University, Foshan 528311, China

**Keywords:** Ag-SnO_2_ composite materials, yield strength, strengthening mechanism, particle-reinforced

## Abstract

Ag-SnO_2_ composites are widely adopted as electrical contact materials in low-voltage apparatuses. Ongoing upgrades of electrical devices impose higher standards for their mechanical strength, machinability, and electrical conductivity, among which the SnO_2_ volume fraction is a dominant factor regulating material performance. In this work, Ag-SnO_2_ electrical contact composites are reinforced with 15 μm SnO_2_ particles at various volume fractions. Increasing SnO_2_ volume fractions can improve the hardness. The ultimate tensile strength reaches a maximum value of 219.1 MPa at the SnO_2_ volume fraction of 18.3 vol%. Excessively high SnO_2_ content (26.5 vol%) leads to the brittle fracture of the composite and a sharp decline in tensile strength. Indirect strengthening dominates the overall mechanical performance, among which grain refinement serves as the primary strengthening mechanism, followed by dislocation multiplication strengthening, while the Orowan looping effect is negligible for coarse 15 μm SnO_2_ particles. This work clarifies the microstructure–performance correlation and strengthening mechanism of particle-reinforced Ag-SnO_2_ composites, providing a theoretical and experimental basis for the optimal design and performance optimization of high-performance electrical contact materials.

## 1. Introduction

Low-voltage electrical contact components rely on silver-based composite materials to sustain frequent cyclic impact, arc thermal shock, and reciprocating friction during long-term service. Sufficient tensile strength, yield strength, and hardness are fundamental to avoid rivet cracking, surface wear, and contact failure, which has positioned the mechanical performance optimization of Ag-SnO_2_ contacts as a critical research topic in electrical material engineering [1,2]. For decades, Ag-CdO composites dominated the low-voltage contact market due to their excellent comprehensive mechanical and electrical properties, yet cadmium toxicity has led to worldwide policy restrictions on cadmium-containing materials [3]. Ag-SnO_2_ has become the most promising eco-friendly alternative, but its mechanical behavior and multi-scale strengthening mechanisms remain less systematically quantified compared with other particle-reinforced metal matrix composites (MMCs), such as Al-based and Cu-based composites [4,5].

Extensive studies on aluminum matrix particle-reinforced composites have established a mature theoretical framework for mechanical strengthening, which can be divided into two core categories: direct load transfer from the matrix to rigid ceramic particles and indirect matrix strengthening, including grain refinement, geometrically necessary dislocation multiplication, and Orowan dislocation bypass [6,7]. In Al-based systems, researchers [8,9] have quantitatively separated the contribution of each strengthening pathway through tensile testing, microstructure statistics, and numerical simulation and clarified the evolution law of the strength, ductility, and fracture mode with increasing reinforcement volume fractions. Generally, the yield strength of MMCs rises monotonically with the reinforcement content, while the ultimate tensile strength peaks at a moderate volume fraction; excessive ceramic particles trigger rapid ductility loss and brittle trans-particle fracture, which severely restricts processing formability [10,11]. This universal mechanical evolution law [12] has been well validated in light alloy matrix composites, while relevant systematic quantitative analysis is still lacking for silver-based electrical contact composites.

For Ag-SnO_2_ composites, existing mechanical research mostly focuses on additives [13] and low volume fraction ranges below 12 wt% SnO_2_ [14]. Similarly to Al matrix composites, adding rigid oxide particles introduces grain boundary pinning effects and generates massive dislocations caused by thermal and elastic modulus mismatch between the two phases, leading to obvious strength enhancement [15,16]. However, few studies [17] adopt unit cell numerical simulation to quantify the independent contributions of load transfer, grain refinement, dislocation hardening, and Orowan strengthening in Ag-based composites.

Moreover, electrical contact materials require a balance between mechanical strength and electrical conductive performance, which creates unique constraints that are distinct from those in structural metal matrix composites [18]. Although a higher SnO_2_ volume fraction improves hardness and wear resistance, the proliferation of two-phase interfaces and interfacial dislocations intensifies electron scattering and reduces conductivity [19]. Few studies systematically record the synchronous variation in tensile properties, hardness, and conductivity across a wide SnO_2_ volume fraction gradient, making it difficult to determine the optimal reinforcement content that satisfies both mechanical processing requirements and electrical service indicators.

Therefore, there is an urgent need to carry out systematic tensile testing, microstructural observation, and finite element numerical simulation to reveal the variation law of the mechanical properties with reinforcement content, decouple the respective contribution of various strengthening mechanisms, and clarify the critical volume fraction corresponding to peak tensile strength and brittle fracture transition, so as to provide a mechanical performance design basis for industrial Ag-SnO_2_ electrical contact materials.

## 2. Experimental Section

### 2.1. Materials and Fabrication

Granular SnO_2_ particles [20] with a uniform particle size of approximately 15 μm were selected as the reinforcing phase. Ag-SnO_2_ composite materials were prepared by hot-pressing the composite powders, synthesized via a heterogeneous precipitation method, at 700 °C and 30 MPa for 1 h, and the detailed process was described in our previous work [21]. Three groups of samples with SnO_2_ volume fractions of 9.5 vol%, 18.3 vol%, and 26.5 vol% (corresponding to mass fractions of 6 wt%, 12 wt%, and 18 wt%) were prepared for a comparative study.

### 2.2. Characterization Methods

The phase composition of the composites was analyzed by X-ray diffraction (XRD; PW 3040/60, PANalytical B.V., Almelo, The Netherlands). The microstructure and particle distribution of the samples were observed by backscattered electron imaging using field emission scanning electron microscopy (FE-SEM; Ultra Plus, Carl Zeiss AG, Oberkochen, Germany) at an acceleration voltage of 15 kV. For Ag grain size characterization, the etchant was prepared by dissolving 2 g of CrO_3_ powder in 100 mL of 1.8 mol/L H_2_SO_4_. The Vickers hardness and electrical conductivity of the composites were tested via a microhardness tester (401MVD, Volpert Measuring Instruments, Shanghai, China) and a vortex conductivity apparatus (FQR 7501A, Xiamen Tianyan Instrument, Xiamen, China), respectively. Vickers microhardness measurements were conducted under a load of 200 gf applied for 8 s, with ten indents placed at 1 mm intervals across each specimen surface to minimize stress field interactions between neighboring impressions. Uniaxial tensile tests were carried out to obtain the true stress–strain curve of pure Ag, using a universal testing machine (AG-X plus, Shimadzu, Kyoto, Japan) at a strain rate of 0.001 s^−1^. The as-sintered Ag-based compacts were sectioned and finish-machined into standard dog-bone tensile specimens—featuring an “I”-shaped reduced section, as detailed in Figure S1—for axial tensile characterization.

### 2.3. Finite Element Simulation Method

**Figure 1:**
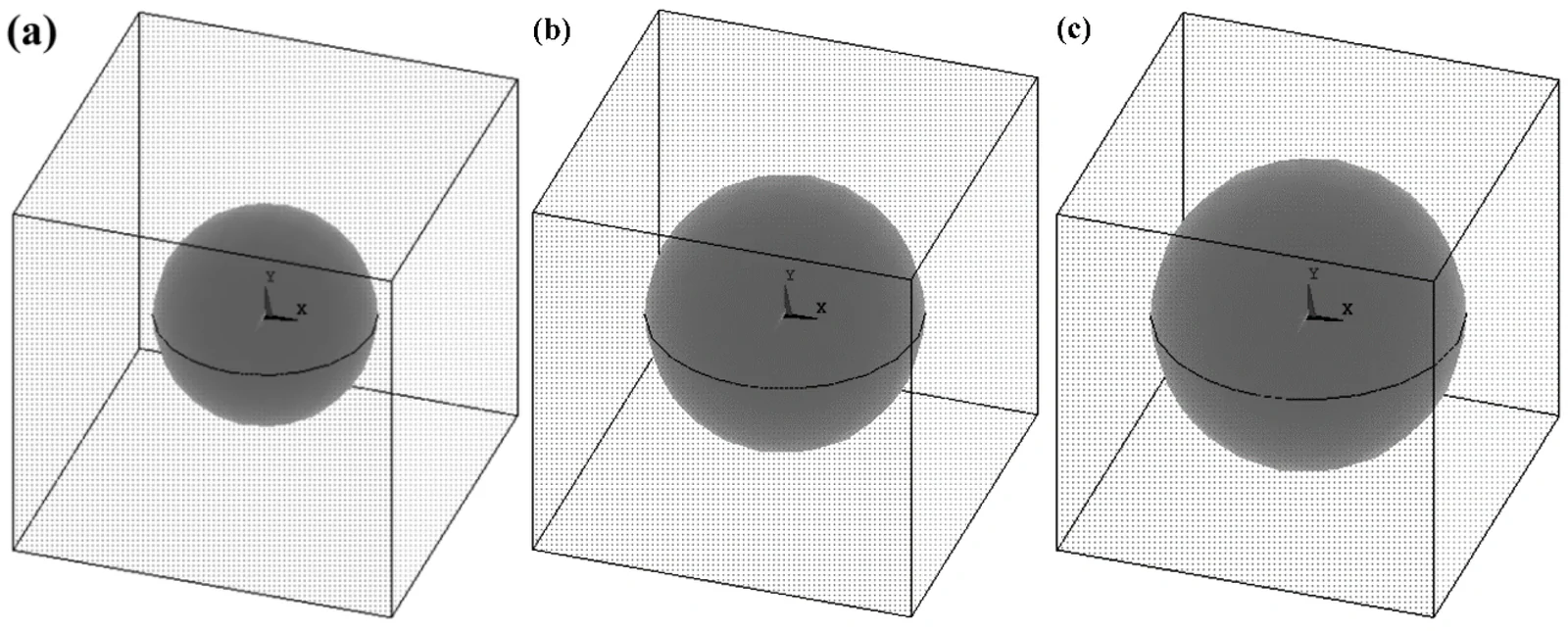
The finite element 3D model of the Ag-SnO_2_ electrical contact materials reinforced by SnO_2_ with different volume fractions: (**a**) 9.5 vol%; (**b**) 18.3 vol%; (**c**) 26.5 vol%.

In this work, the ANSYS structural analysis software (V17.0) was employed to conduct finite element simulations to determine the tensile mechanical behavior and internal stress evolution of SnO_2_ particle-reinforced Ag matrix composites. A top-down solid modeling strategy was adopted in the numerical calculation. Firstly, cubic matrix units and spherical reinforcement primitives were established. To balance calculation efficiency and numerical accuracy, a three-dimensional single-sphere unit cell model was adopted for all simulation cases, as shown in Figure 1. To guarantee the continuity and periodicity of the composite microstructure, coupled boundary conditions were applied on the four side planes parallel to the tensile direction to limit the in-plane displacement and maintain planar flatness. A uniaxial displacement load was applied on the upper and lower surfaces perpendicular to the tensile direction. The whole model was meshed with a uniform mesh size for consistent calculation precision.

The accuracy of finite element simulation strongly relies on the authentic mechanical response of the pure Ag matrix. In this study, pure silver specimens were hot-pressed at 700 °C for 1 h under 30 MPa pressure and machined into standard tensile specimens. As shown in Figure S1, the measured elastic modulus, yield strength, ultimate tensile strength, and elongation of the as-prepared pure Ag control (Figure S1 in Supplementary Materials) are 82.6 GPa, 51.2 MPa, 105.8 MPa, and 56.8%, respectively. The elastic modulus of silver closely approximates that of pure Ag reported in the literature [22], thereby validating the reliability of the measured stress–strain curve. Consequently, the experimentally obtained curve was employed for subsequent simulations. The basic physical parameters of the Ag matrix and the SnO_2_ reinforcement used in finite element calculation are listed in Table 1. Considering the ultra-high strength and brittle ceramic characteristics of SnO_2_ particles, SnO_2_ was simplified as an ideal linear elastic material without plastic deformation during the whole simulation process.

## 3. Results and Discussion

### 3.1. Phase Composition and Microstructure of Ag-SnO_2_ Composites

**Figure 2:**
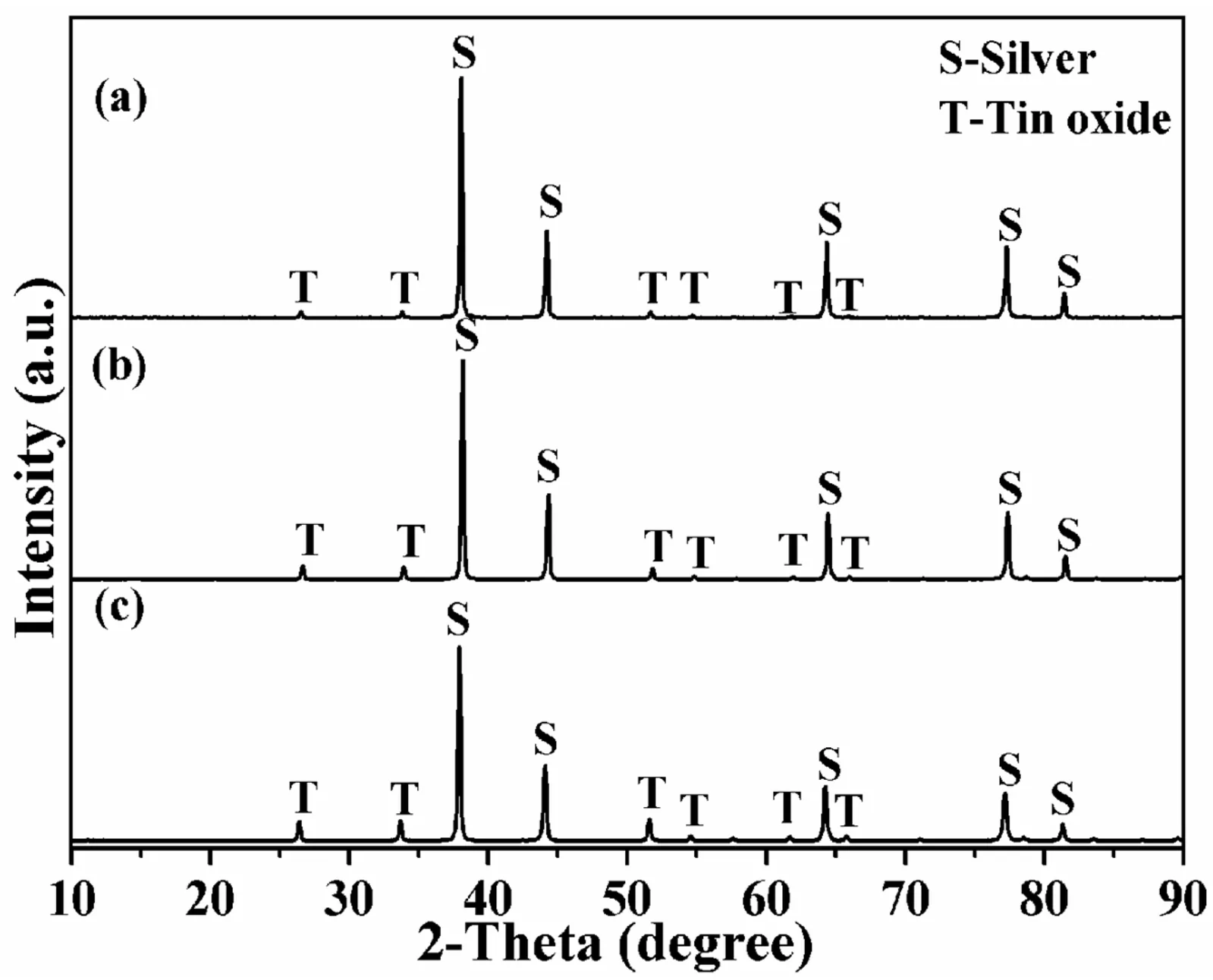
XRD patterns of the Ag-SnO_2_ electrical contact materials reinforced by SnO_2_ with different volume fractions: (**a**) 9.5 vol%; (**b**) 18.3 vol%; (**c**) 26.5 vol%.

The XRD patterns of Ag-SnO_2_ composites with different SnO_2_ volume fractions are displayed in Figure 2. For all samples, we only observed the characteristic diffraction peaks of cubic metallic Ag and tetragonal SnO_2_, with no impurity peaks detected. With the increase in the SnO_2_ volume fraction, the relative intensity of the SnO_2_ diffraction peaks increased gradually relative to the Ag matrix peaks, which was consistent with the variation in reinforcement content.

**Figure 3:**
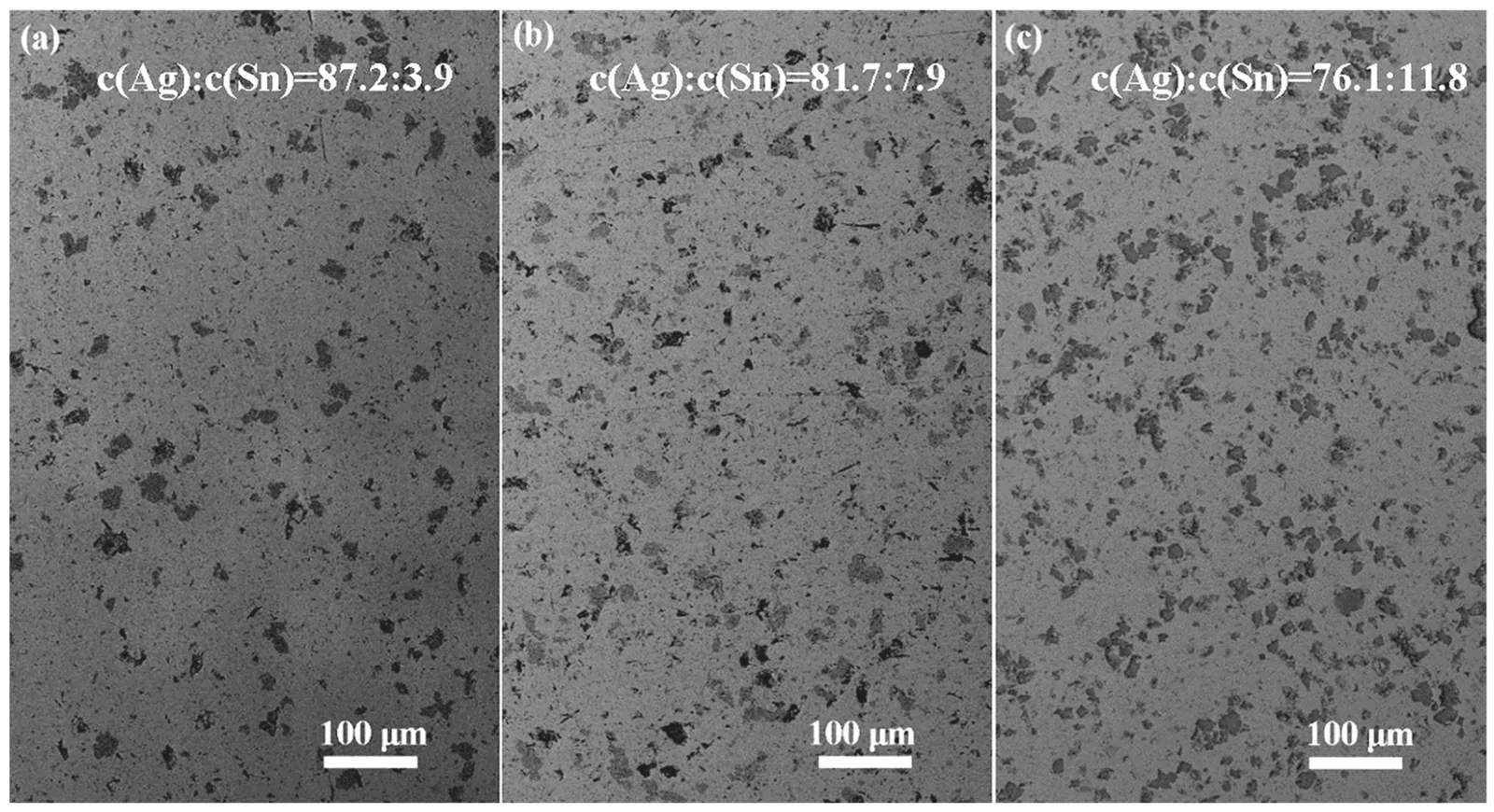
Backscattered electron images of the Ag-SnO_2_ electrical contact materials reinforced by SnO_2_ with different volume fractions: (**a**) 9.5 vol%; (**b**) 18.3 vol%; (**c**) 26.5 vol%. Inset: corresponding EDS results.

The backscattered electron micrographs of the composites are shown in Figure 3. For all samples, SnO_2_ particles are uniformly dispersed in the Ag matrix. As the SnO_2_ volume fraction increases, the average spacing between reinforcing particles decreases gradually. An energy-dispersive X-ray spectroscopy (EDS) analysis was used to confirm the actual SnO_2_ content in the composites, and the results are labeled in Figure 3. The measured atomic ratio of Ag–Sn agrees well with the designed composition.

**Figure 4:**
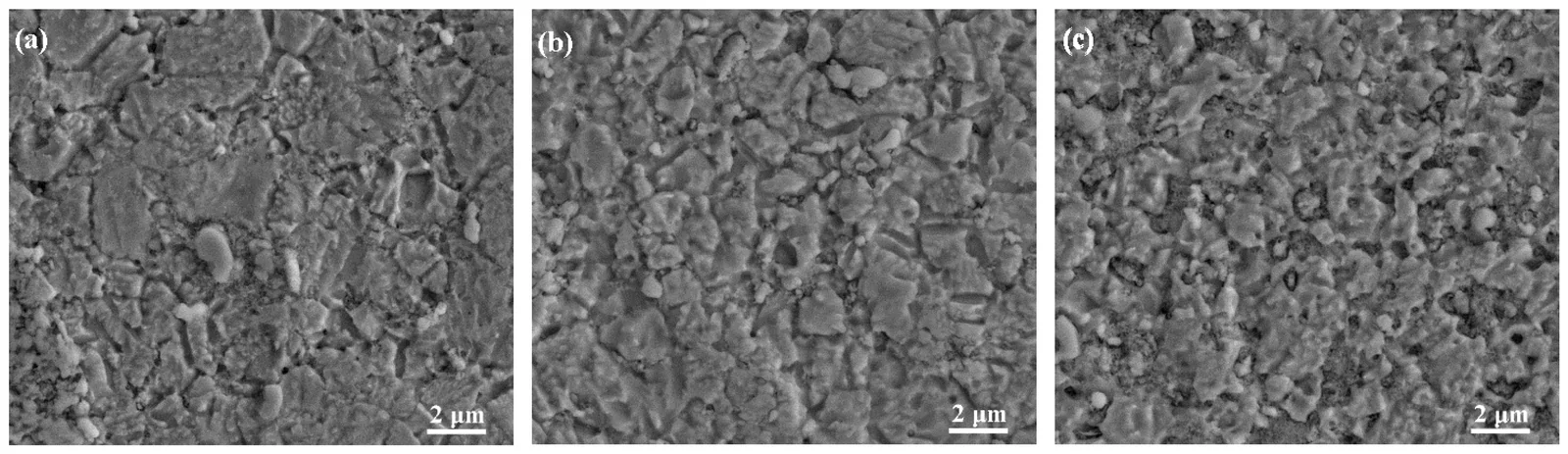
Microstructures of the Ag-SnO_2_ electrical contact materials reinforced by SnO_2_ with different volume fractions: (**a**) 9.5 vol%; (**b**) 18.3 vol%; (**c**) 26.5 vol%.

The microscopic morphologies of the Ag matrix grains of composites with different SnO_2_ content are shown in Figure 4. According to the statistical analysis of the SEM images, the average grain sizes of the Ag matrix in the composites containing 9.5 vol%, 18.3 vol%, and 26.5 vol% SnO_2_ are 2.3 μm, 1.2 μm, and 1.0 μm, respectively. SnO_2_ particles pinned at Ag grain boundaries inhibit grain boundary migration and grain growth, resulting in obvious grain refinement.

### 3.2. Physical Properties of Ag-SnO_2_ Composites

**Figure 5:**
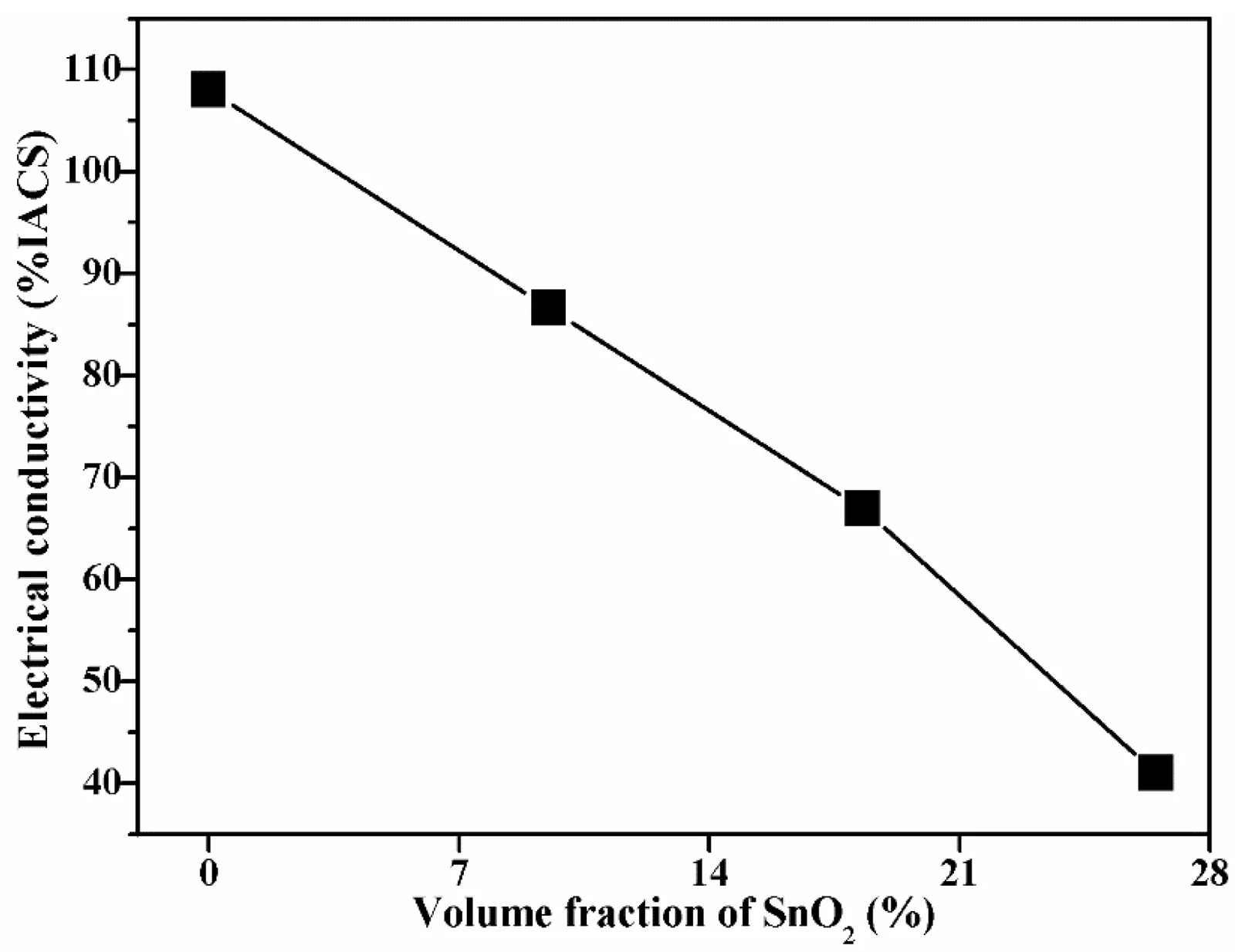
The effect of the SnO_2_ volume fraction on the electric conductivity of Ag-SnO_2_ contact materials.

**Figure 6:**
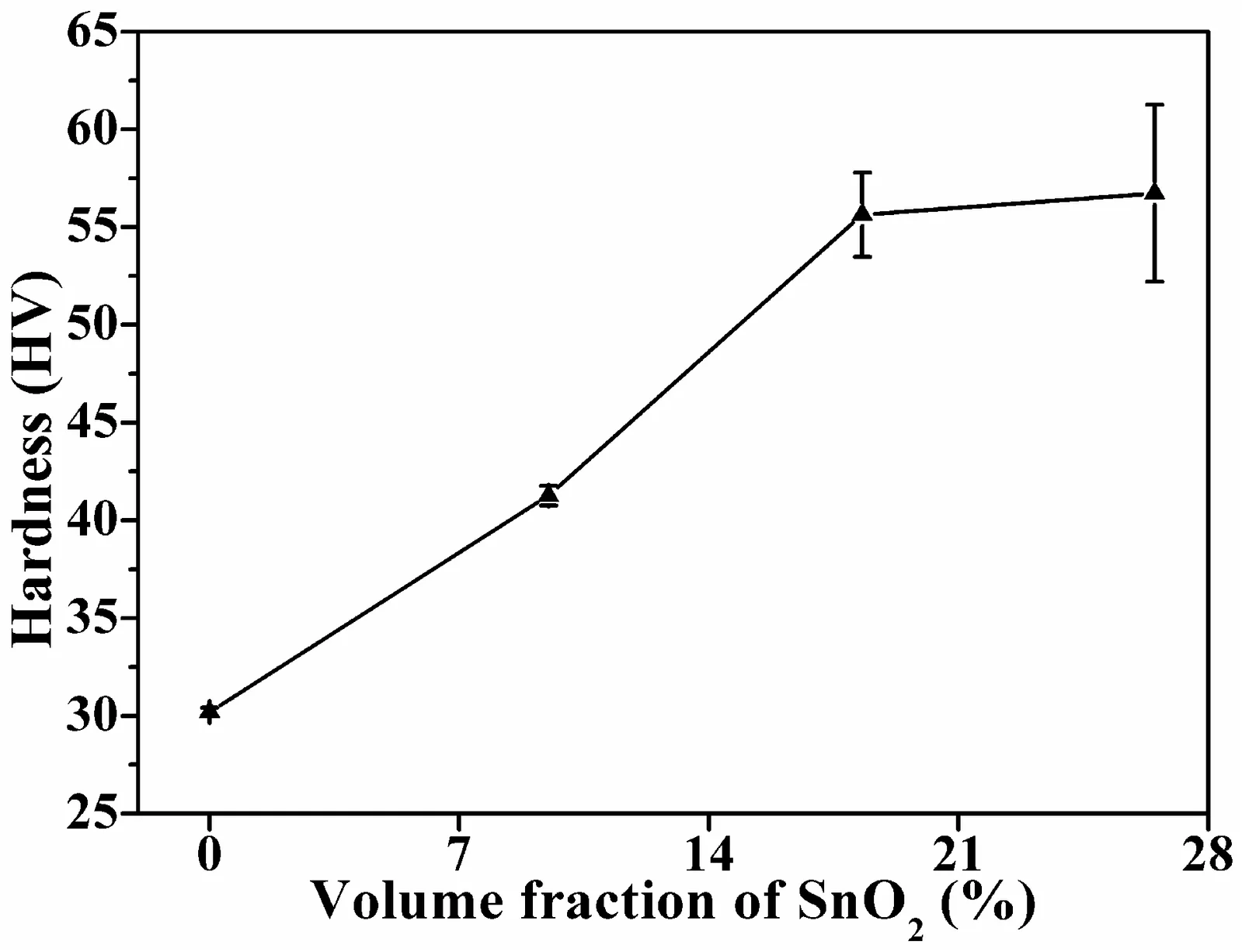
The effect of the SnO_2_ volume fraction on the hardness of Ag-SnO_2_ contact materials.

As shown in Table 2, both the Archimedes density and the theoretical density of the sintered Ag-SnO_2_ composites decrease with increasing SnO_2_ volume fractions. The introduction of SnO_2_ reinforcements may induce additional porosity in the composite. As shown in Figure 5, the electrical conductivity of the Ag-SnO_2_ composites decreases with increasing SnO_2_ volume fractions. Two dominant electron scattering mechanisms account for the conductivity degradation. The total interfacial area between Ag and SnO_2_ increases with increasing reinforcement volume fractions, which intensifies interfacial electron scattering, resulting in a continuous decrease in electrical conductivity. The variation in composite hardness is shown in Figure 6. The hardness increases significantly as the SnO_2_ volume fraction rises from 0 vol% (pure Ag) to 18.3 vol%, while further increasing the SnO_2_ content brings a negligible hardness improvement. On one hand, a high SnO_2_ fraction hinders uniform dispersion, and some SnO_2_ agglomeration can be observed in Figure 3c. Particle agglomeration reduces the number of effective interfaces for load transfer. On the other hand, the large error bar in hardness for samples with high SnO_2_ fractions (Figure 6) indicates deteriorating microstructural homogeneity. Meanwhile, the relative density decreases continuously with increasing reinforcement content (Table 2). The resulting pores act as micro-defects, offsetting the potential hardening contribution from additional SnO_2_ particles. Under the combined influence of particle agglomeration, a non-uniform microstructure, and a reduced density, the hardness plateaus at excessive SnO_2_ volume fractions.

**Figure 7:**
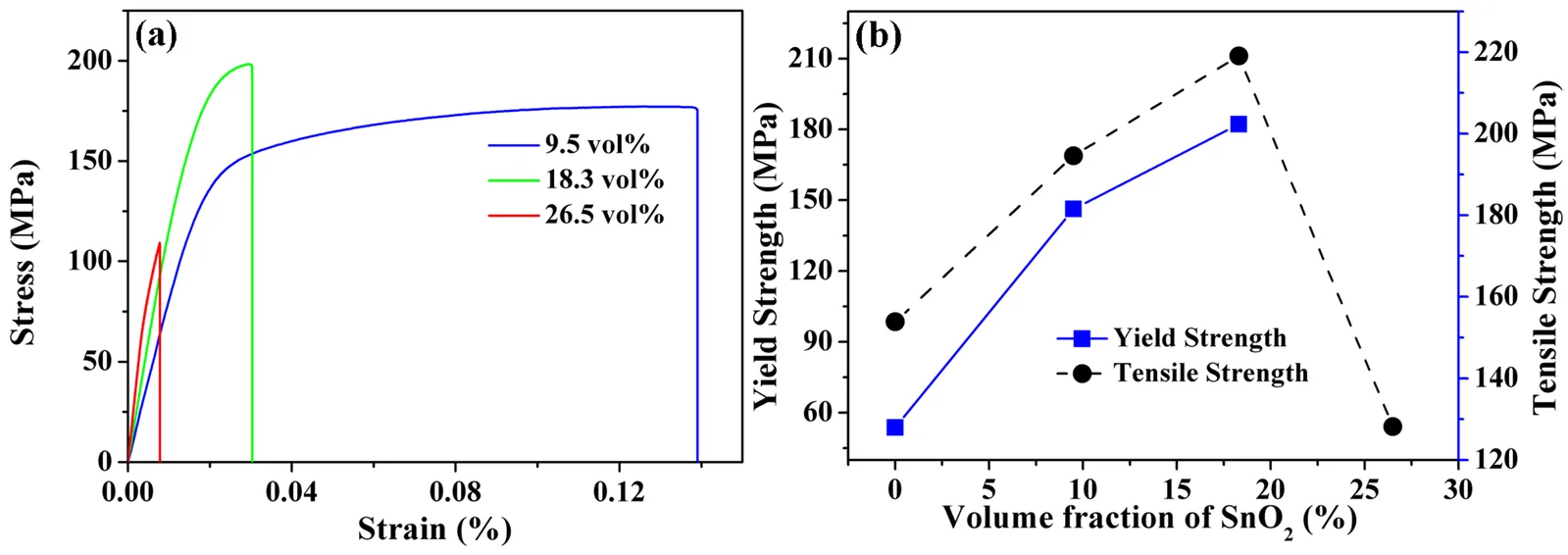
(**a**) The stress–strain curves of the Ag-SnO_2_ electrical contact materials reinforced by SnO_2_ with different volume fractions; (**b**) the effect of the SnO_2_ volume fraction on the mechanical properties of the Ag-SnO_2_ contact materials.

The mechanical properties of Ag-SnO_2_ composites with different SnO_2_ volume fractions are presented in Figure 7. The yield and tensile strength of the as-prepared pure Ag are 51.2 and 105.8 MPa, respectively. With increasing SnO_2_ volume fractions (0~18.3%), the yield strength of the composites increases monotonically. When the SnO_2_ volume fraction reaches 9.5 vol% and 18.3 vol%, the tensile strength increases significantly to 194.6 MPa and 219.1 MPa, respectively. However, when the SnO_2_ content increases further to 26.5 vol%, the composite exhibits typical brittle fracture characteristics without obvious plastic deformation, and the tensile strength drops sharply to 128.2 MPa.

**Figure 8:**
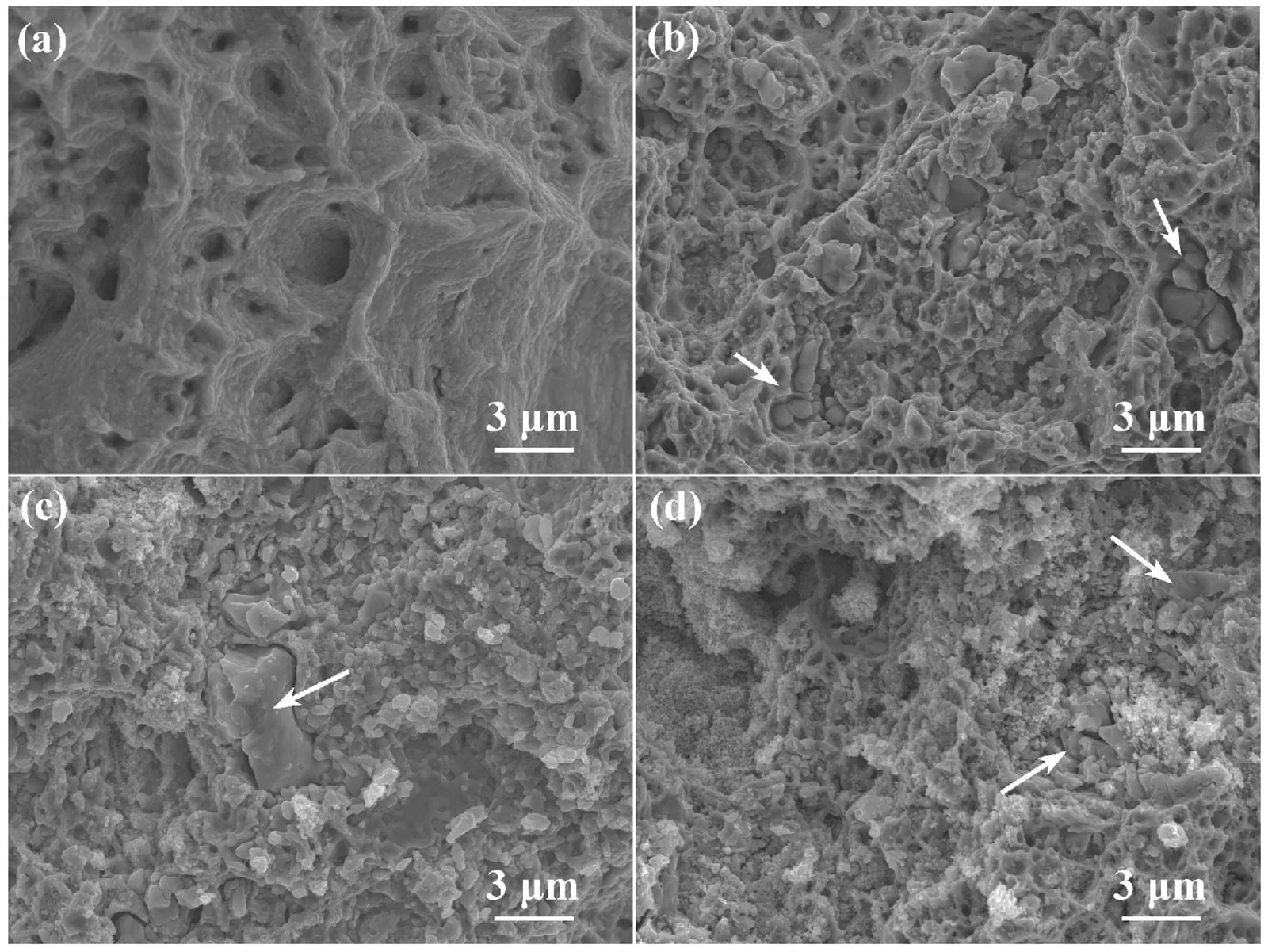
The fracture surface images of Ag-SnO_2_ electrical contact materials with various volume fractions of SnO_2_: (**a**) 0, (**b**) 9.5 vol%, (**c**) 18.3 vol%, (**d**) 26.5 vol%. Arrows indicate fragmented SnO_2_ reinforcements.

The fracture surfaces of the tensile-tested specimens were examined, as shown in Figure 8. The pure silver sample displayed numerous dimples measuring 1–3 μm in diameter, characteristic of ductile fracture. As the SnO_2_ volume fraction increases, the dimple dimensions in the composite samples progressively decrease, signifying a gradual transition from ductile to brittle fracture. For the sample reinforced with 26.5 vol% SnO_2_, the fracture surface exhibits typical brittle characteristics with cleavage facets and minimal plastic deformation, marking the onset of severe brittle fracture.

### 3.3. Strengthening Mechanism of Ag-SnO_2_ Composites

The mechanical property improvement of particle-reinforced metal matrix composites is jointly determined by direct and indirect strengthening mechanisms. Direct strengthening refers to load transfer from the soft Ag matrix to rigid SnO_2_ particles, while indirect strengthening includes grain refinement, dislocation multiplication, and Orowan bypass strengthening. The quantitative contribution of each mechanism was analyzed via finite element simulation and theoretical calculation.

#### 3.3.1. Direct Load Transfer Strengthening

**Figure 9:**
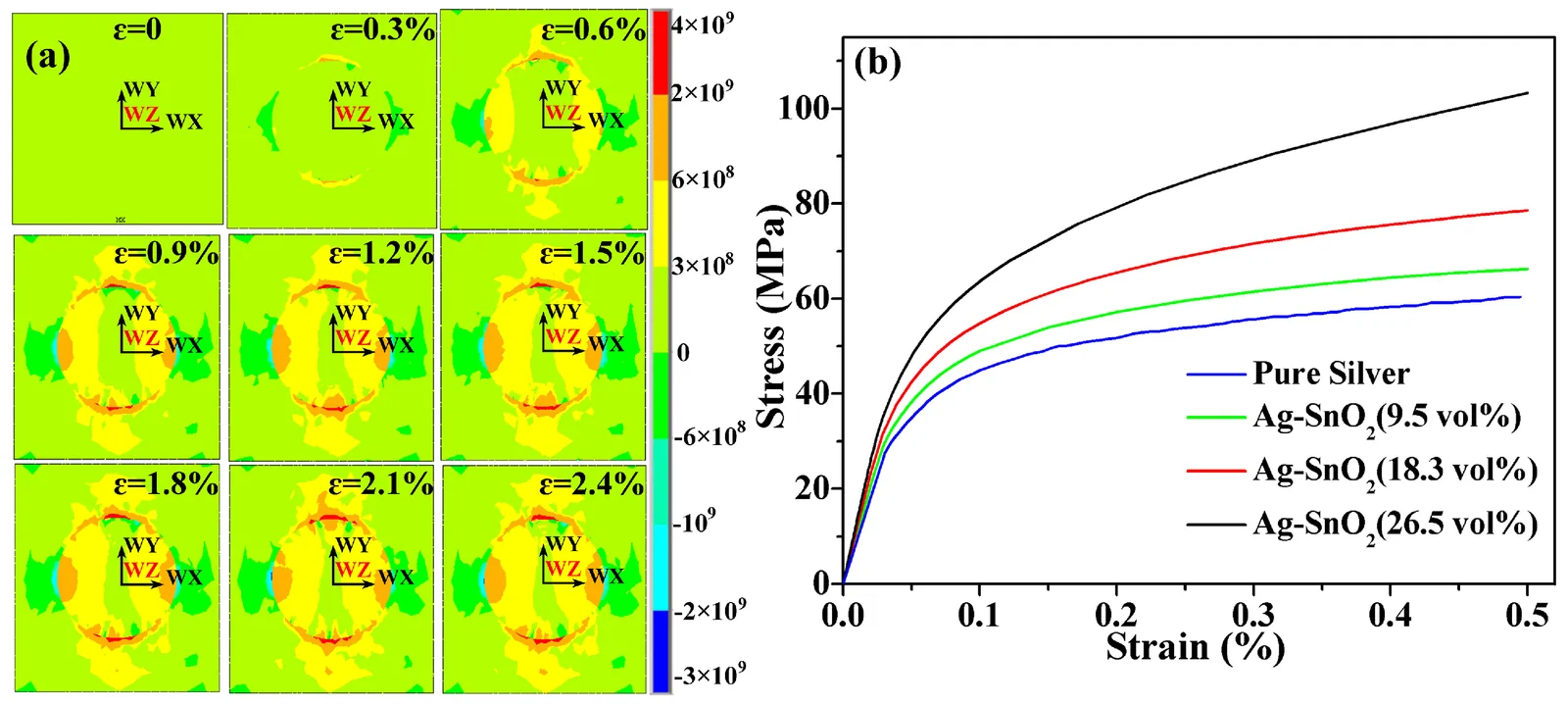
(**a**) Simulated stress nephograms of Ag-SnO_2_ (9.5 vol% SnO_2_) electrical contact materials under different tensile strains and (**b**) simulated stress–strain curves of Ag-SnO_2_ composites with different SnO_2_ volume fractions under the single direct strengthening mechanism.

The load transfer strengthening behavior was quantitatively investigated using the finite element simulation method established in Section 2.3. Taking the 9.5 vol% SnO_2_ composite as a typical case, the sequential stress distribution evolution under continuous tensile strain is presented in Figure 9a. Significant stress concentration occurs at the Ag/SnO_2_ interface, and rigid SnO_2_ particles bear a partial tensile load transferred from the Ag matrix. The simulated stress–strain curves for independent direct load transfer strengthening are shown in Figure 9b. The calculated yield strength increments induced by pure load transfer amount to 5.0 MPa, 14.3 MPa, and 30.3 MPa for composites reinforced with 9.5 vol%, 18.3 vol%, and 26.5 vol% SnO_2_, respectively. Compared with the experimental tensile results, the strengthening contribution of direct load transfer is extremely limited. For the coarse 15 μm SnO_2_ particles used in this work, the effective load-bearing capacity of the reinforcements is insufficient, demonstrating that direct strengthening is not the dominant reinforcing mechanism in the fabricated Ag-SnO_2_ composites.

#### 3.3.2. Indirect Load Transfer Strengthening

The addition of SnO_2_ significantly changes the microstructural characteristics of the Ag matrix, producing multiple indirect strengthening effects. Indirect strengthening mechanisms include grain boundary strengthening via Hall–Petch refinement, strain hardening arising from dislocation multiplication and entanglement, and Orowan strengthening. In this section, these three typical indirect strengthening mechanisms are quantitatively calculated and decoupled based on classical composite strengthening theories and experimental microstructure parameters.

Grain refinement strengthening follows the classic Hall–Petch formula, which is the primary strengthening mechanism of the prepared Ag-SnO_2_ composites [23]:

(1) $$\sigma_{g}=\sigma_{0}+\frac{k_{y}}{\sqrt{d_{m}}}$$

where *σ*_g_ is the yield strength of the pure silver matrix, *σ*_0_ is the yield strength of a pure silver single crystal (24 MPa [24]), *d*_m_ is the grain size of the silver matrix, and *k*_y_ is the Hall–Petch slope constant of pure silver (1.69 MPa·cm^1/2^ [24]). Based on the Hall–Petch equation and the average grain size data summarized in Table S1, the predicted strength increments attributable to grain refinement in Ag-SnO_2_ composites containing 9.5 vol%, 18.3 vol%, and 26.5 vol% SnO_2_ are 82.3 MPa, 123.2 MPa, and 135.8 MPa, respectively.

In Ag-SnO_2_ composites, the significant differences in the elastic modulus and thermal expansion coefficient between rigid SnO_2_ particles and the ductile Ag matrix produce prominent strain mismatch at the phase interfaces, generating abundant geometrically necessary dislocations (GNDs) and statistically stored dislocations (SSDs) and resulting in dislocation strengthening. The yield strength enhancements induced by these two types of dislocations can be quantitatively evaluated according to the Taylor dislocation strengthening relationship reported in the literature [23].

In addition, the Orowan bypass strengthening effect was calculated based on the modified Orowan equation [25]. The results show that the strength increment caused by the Orowan mechanism is only 1.3–2.1 MPa for all samples. The negligible strengthening effect is attributed to the large size of the SnO_2_ particles, which leads to large interparticle spacing and a weak interaction between particles and dislocations.

#### 3.3.3. Coupled Mixed Strengthening Model

**Figure 10:**
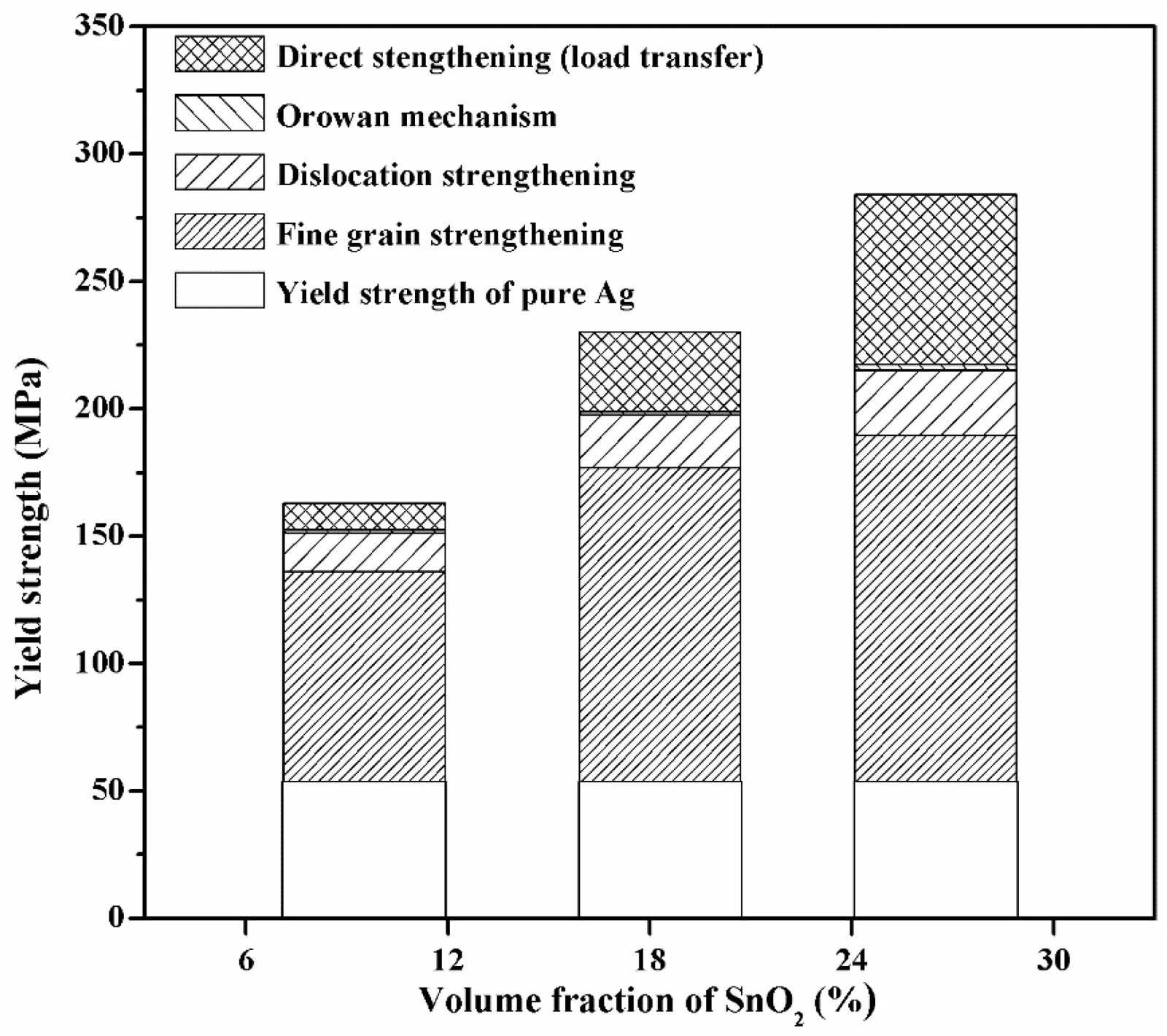
The simulated yield strength of Ag-SnO_2_ electrical contact materials reinforced by SnO_2_ with the different volume fraction contributions of each mechanism.

The macroscopic mechanical properties of Ag-SnO_2_ composites arise from the synergistic coupling of all strengthening mechanisms. The modified matrix yield strength considering multiple indirect strengthening effects was substituted into the finite element load transfer model to establish a mixed strengthening prediction model. The contribution proportions of each strengthening mechanism are displayed in Figure 10. The simulated yield strength of the mixed model is highly consistent with the experimental data. The slight deviation between the simulation and experiment is caused by interfacial debonding and the local fracture of SnO_2_ particles during tensile deformation, which partially releases local stress concentration and thereby reduces the macroscopic strength of the composites.

## 4. Conclusions

In this work, Ag-SnO_2_ electrical contact composites with different SnO_2_ volume fractions were fabricated. With increasing SnO_2_ volume fractions, the hardness of the composites increases continuously, while the electrical conductivity decreases. For SnO_2_ content ranging from 0 to 18.3 vol%, the composite yield strength increases monotonically with increasing SnO_2_ content. The ultimate tensile strength first increases and then decreases, with optimal comprehensive mechanical performance obtained at 18.3 vol% SnO_2_, reaching the maximum tensile strength of 219.1 MPa. An excessively high SnO_2_ volume fraction (26.5 vol%) induces the brittle fracture of the composites, resulting in a sharp decline in tensile strength and no significant improvement in hardness. The mechanical reinforcement of Ag-SnO_2_ composites depends on the synergistic effect of direct and indirect strengthening mechanisms. Indirect strengthening dominates the mechanical performance, with grain refinement being the primary mechanism, dislocation multiplication being the secondary mechanism, and the Orowan strengthening effect being negligible for the 15 μm coarse SnO_2_ particle reinforcements.

## Figures and Tables

**Table 1:** Physical parameters used for simulation [22].

Material	Young’s Modulus (GPa)	Density (g/cm^3^)	Poisson’s Ratio
Ag	82.6	10.49	0.38
SnO_2_	328	6.38	0.4

**Table 2:** Density of Ag-SnO_2_ electrical contact materials reinforced by SnO_2_ with different volume fractions.

SnO_2_ Volume Fraction (%)	Theoretical Density (g/cm^3^)	Archimedes Density (g/cm^3^)	Relative Density (%)
0	10.49	10.41	99.2
9.5	10.10	9.90	98.0
18.3	9.74	9.50	97.5
28.5	9.40	9.14	97.4

## Data Availability

The original contributions presented in this study are included in the article/Supplementary Materials. Further inquiries can be directed to the corresponding authors.

## Supplementary Materials

The following supporting information can be downloaded at https://www.mdpi.com/article/10.3390/ma19163545/s1. Figure S1. Schematic diagram of the “I-shape” sample workpiece. Figure S2. Stress–strain curve of the identically processed Ag control hot-pressed at 700 °C and 30 MPa for 1 h. Figure S3. Three-dimensional models of different mesh sizes: (a) mesh size 1 and (b) mesh size 3. Figure S4. (a) The stress–strain curves of the composite and (b) the yield strength of the composite under different mesh sizes. Figure S5. Microstructure of the pure Ag reference. Table S1. Average Ag matrix grain sizes of Ag-SnO_2_ composites with different SnO_2_ fractions.

## References

[1] Gao K., Wang H.J., Li H.Y., Cao Q.G., Li J. (2025). Enhancement of arc erosion and welding resistance in Ag-SnO_2_ contacts through WO_3_ and Ag_2_WO_4_ additives. Adv. Eng. Mater..

[2] Mao T.C., Wang J., Wang Z., Fang C., Mao Z.S., Lv H.P., Zhang S., Chang Y.L., Zhu J.B., Kuang B. (2025). Towards understanding the synergistic reinforcement of erosion and impact resistance mechanisms in Ag-SnO_2_-CuO contacts by composite nanofibres. Ceram. Int..

[3] Wang M., Chen Z.F., Song W., Hong D.Z., Huang L., Li Y.H. (2021). A review on cadmium exposure in the population and intervention strategies against cadmium toxicity. Bull. Environ. Contam. Toxicol..

[4] Xia Y.X., Sun Y.T., Zhu P., Li W.T., Hu J.R., Hao L.L., Liu Y., Ju B.Y., Yang W.S., Wu G.H. (2025). Revealing the compatible deformatoin mechanism of metal particles reinforced aluminum matrix composites. Mat. Sci. Eng. A-Struct..

[5] Tang J.C., Omar M.Z., Jiang R.P., Mohamed I.F. (2025). Effect of TiB_2_ on the Microstructure and Mechanical Properties of TiB_2_/Al-5Cu Matrix Composites. JOM.

[6] Jiang M.Y., Zhang X.Z., Mei H., Xu S., Liu L.S. (2024). The coupled effects of grain boundary strengthening and orowan strengthening examined by dislocation dynamics simulations. Comput. Mater. Sci..

[7] Shen C.Y., Ye Y.Q., Chen F., Zhuo Y.M., Zhang J.M., Mao J.W., Le J.W., Han Y.F., Lu W.J. (2026). Evading strength-ductility trade-off in titanium matrix composites via high-critical resolved shear stress dislocation promotion and grain subdivision. Rare Met..

[8] Liu Y., Zan Y.N., Wang D., Song M., Shao X.H., Hao H.L., Jin Q.Q., Wang W.G., Xiao B.L., Ma Z.Y. (2024). Temperature mechanical properties: Multimechanism synergistic strengthening strategy. Nano Lett..

[9] Chen Y., Jian Z.Y., Ren Y.M., Li K., Dang B., Guo L.X. (2023). Influence of TiB_2_ volume fraction on SiC_p_/AlSi10Mg composites by LPBF: Microstructure, mechanical, and physical properties. J. Mater. Res. Technol..

[10] Gao X., Lu X.N., Zhang X.X., Qian M.F., Li A.B., Geng L., Wang H., Liu C., Ouyang W.T., Peng H.X. (2024). Effect of particle strength on SiCp/Al composite properties with network architecture design. Materials.

[11] Qayyum F., Chiu C.C., Tseng S., Rustamov U., Berndorf S., Shen F.H., Guk S., Chao C.K., Prahl U. (2024). Local strain heterogeneity and damage mechanisms in zirconia particle-reinforced TRIP steel MMCs: In situ tensile testing with digital image processing. J. Mater. Sci..

[12] Hussein O., Mishin Y. (2026). Self-pinning mechanism for grain boundary stabilization. Acta Mater..

[13] Qi D.R., Li G.J., Han X.L., Fang X.Q., Feng W.J., Tian R.L. (2023). Effect of the in-situ formed CuO additive on the fracture behaviour and failure mechanism of Ag-SnO_2_ composites. Eng. Fract. Mech..

[14] Qi Z.X., Li L.H., Yi H., Liang W.G., Liu J.Q., Wei M.C. (2025). Effect of SnO_2_ particulate characteristics on mechanical properties of Ag/SnO_2_ electrical contact materials. Int. J. Solids Struct..

[15] Zhou Y.J., Zhou J.Y., Zhang W.W., Fan J.L., Wu S., Li X.Q., Wei S.Z. (2025). Improvement the thermal stability and mechanical properties of ODS Cu alloy by forming composite oxide dispersion. Mater. Charact..

[16] Liu K., Su Y.S., Wang X.Z., Cai Y.P., Cao H., Ouyang Q.B., Zhang D. (2023). Achieving simultaneous enhancement of strength and ductility in Al matrix composites by employing the synergetic strengthening effect of micro- and nano-SiCps. Compos. Part B-Eng..

[17] Fu X.Q., Liang L.H., Wei Y.G. (2019). Modeling of atomistic scale shear failure of Ag/MgO interface with misfit disocation network. Comput. Mater. Sci..

[18] Ding Y.H., Jiang Q.W., Lin H.Z., Liu X.Y., Zhou J.D., Tang D.L., Yi J.J., Feng J. (2025). Laminated architecture with interpenetrating 3D-Gr enhancement of strength and electrical conductivity in Cu matrix composites. J. Alloys Compd..

[19] Wang J., Li D.M., Wang Y.P. (2014). Microstucture and properties of Ag-SnO_2_ materials with high SnO_2_ content. J. Alloys Compd..

[20] Lin Z.J., Liang Y.C., Sang Y.H., Chen S., Chen X., Sun X.D., Liu H., Dai P.Q. (2020). Morphology-dependent highly active microcrystalline stannous oxalate photocatalysts with selectively exposed facets and low specific surface areas. Appl. Surf. Sci..

[21] Lin Z.J., Liang Y.C., Zeng Y.M., Chen X., Liu M.M., Dai P.Q., Chen J.L., Sun X.D. (2022). Morphology-tunable synthesis and formation mechanism of SnO_2_ particles and their application in Ag-SnO_2_ electrical contact materials. Ceram. Int..

[22] Cao L., Yao J., Qian S.G., Huang J.F. (2010). Stress-fields numerical simulation of particle reinforced Ag matrix composite material. Sci. Techol. Eng..

[23] Dai L.H., Ling Z., Bai Y.L. (2001). Size-dependent inelastic behavior of particle-reinforced metal-matrix composites. Compos. Sci. Technol..

[24] Aldrich J.W., Armstrong R.W. (1970). The grain size dependence of the yield, flow an fracture stress of commercial purity silver. Metall. Trans..

[25] Schmid E., Walter B. (1935). Kristallplastizitāt: Mit Besonderer Berücksichtigung der Metalle.

