# Supplementary material for: Microstructure and Properties of Ag-SnO2 Electrical Contact Composites with Different SnO2 Volume Fractions
Source: Materials (Basel). 2026 Aug 21;19(16):3545. doi: 10.3390/ma19163545 (PMC13514477; doi:10.3390/ma19163545)
Supplement: Supplementary file 1 [file materials-19-03545-s001.zip › materials-4495509-supplementary.pdf]

Supplementary Materials

Microstructure and Properties of Ag-SnO_2 Electrical Contact Composites with Different SnO_2 Volume Fractions

Zhijie Lin [1,*], Bin Liu [1] and Xudong Sun [2,3,*]

[1] College of Materials Science and Engineering, Fujian University of Technology, Fuzhou 350118, China
[2] School of Materials Science and Engineering, Northeastern University, Shenyang 110819, China
[3] Foshan Graduate School of Innovation, Northeastern University, Foshan 528311, China
* Correspondence: zjlin@fjut.edu.cn (Z.L.); xdsun@mail.neu.edu.cn (X.S.)

The as-sintered Ag-based compacts were sectioned and finish-machined into standard dog-bone tensile specimens—featuring the "I"-shaped reduced section detailed in Figure S1—for axial tensile characterization.

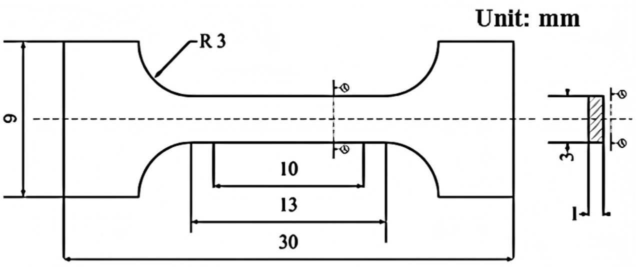

Figure S1. Schematic diagram of the “I-shape” sample workpiece.

The intrinsic matrix stress–strain curve is critical for the finite element analysis of metal matrix composites. In this study, the pure Ag curve was obtained experimentally. Silver powder was placed in a graphite die and then hot-pressed at 700 °C for 1 hour under pressure of 30 MPa. After cooling, the compact was machined into tensile specimens. Uniaxial tensile tests were performed to obtain the stress–strain curve of pure Ag. The as-sintered specimen achieved a relative density of 99.2%. The measured elastic modulus was 82.6 GPa. The yield strength was 51.2 MPa. The ultimate tensile strength was 105.8 MPa. The elongation to fracture was 53.4%. The measured elastic modulus aligns closely with the literature value for pure Ag [22]. This validates the experimental curve. Therefore, the experimentally obtained stress–strain curve (Figure S2) was adopted for all simulations.

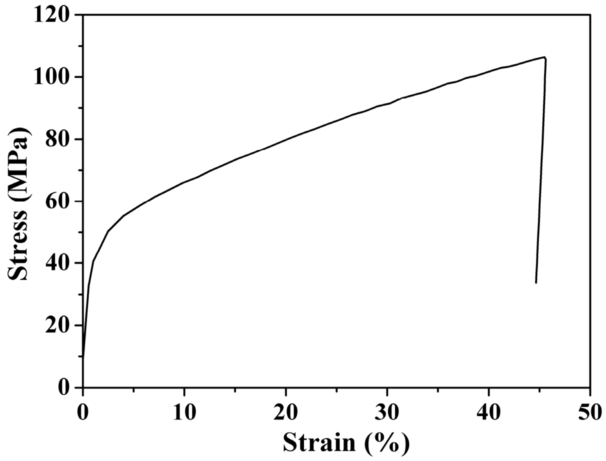

Figure S2. Stress–strain curve of the identically processed Ag control hot-pressed at 700 °C and 30 MPa for 1 h.

A three-dimensional RVE model was established. The matrix was silver (Ag). The particle was tin dioxide (SnO_2). The particle volume fraction was 18.3%. The model contained a single SnO_2 particle. Two mesh densities were employed: Level 1 and Level 3 (Figure S3). Level 1 is finer than Level 3. Figure S4 presents the stress–strain curves, yield strengths, and stresses at 1% strain for both mesh densities. The mesh density significantly affects the results. Level 3 predicts higher stresses than Level 1. This deviation intensifies with increasing strain. To eliminate mesh-induced numerical errors, all subsequent simulations adopted SmartSize Level 1. A limitation remains. The single-particle periodic RVE cannot capture true particle irregularity or random distribution. This simplified model serves to investigate fundamental matrix–particle interfacial load transfer.

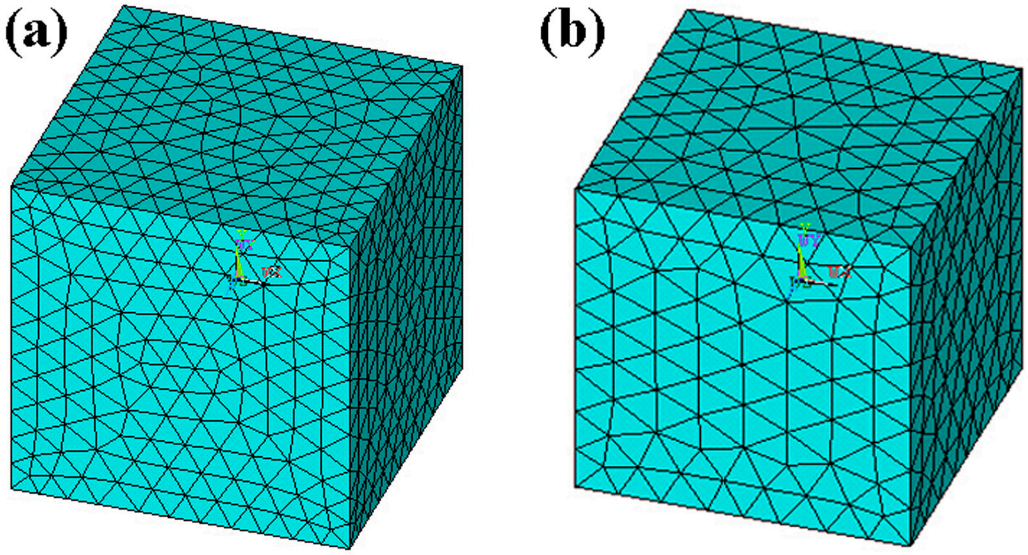

Figure S3. Three-dimensional models of different mesh sizes: (a) mesh size 1 and (b) mesh size 3.

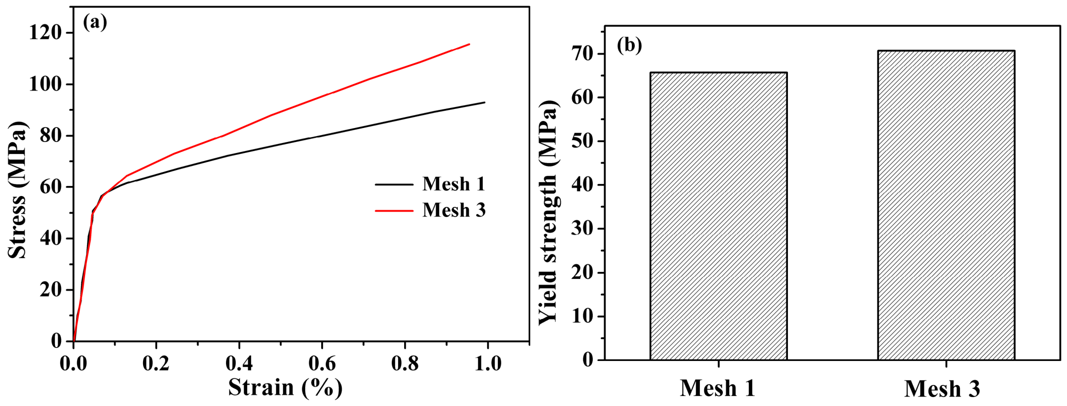

Figure S4. (a) The stress–strain curves of the composite and (b) the yield strength of the composite under different mesh sizes.

The as-prepared pure Ag reference specimen was metallographically etched and subsequently examined. The microstructure of the pure Ag sample is presented in Fig. S5, whereas that of the composite material is shown in Fig. 4. The average grain sizes of the fabricated samples, determined by statistical analysis, are summarized in Table S1.

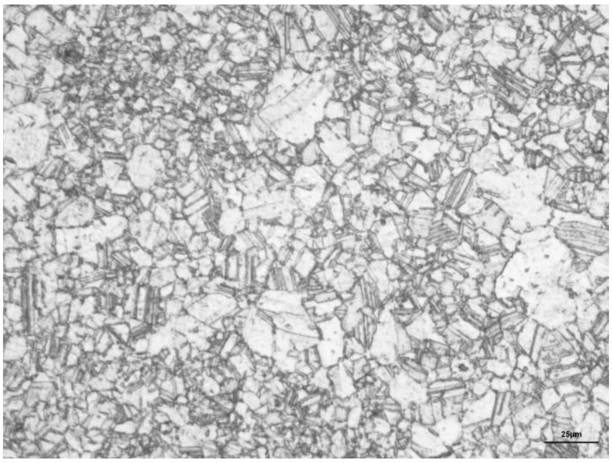

Figure S5. Microstructure of the pure Ag reference.

Table S1. Average Ag matrix grain sizes of Ag-SnO_2 composites with different SnO_2 fractions.

SnO_2 volume fraction (%)	0	9.5	18.3	26.5
Average Ag matrix grain size (μm)	17.86	2.22	1.18	1.01
